# Attitudinal, Behavioral, and Environmental Correlates of Child and Parent Self-Efficacy in Walking to School

**DOI:** 10.3390/ijerph14121588

**Published:** 2017-12-17

**Authors:** Young-Jae Kim, Chanam Lee, Wenhua Lu, Jason A. Mendoza

**Affiliations:** 1 Department of Forest Resources and Landscape Architecture, Yeungnam University, 280 Daehak-Ro, Gyeongsan, Gyeongbuk 38541, Korea; 2 Department of Landscape Architecture and Urban Planning, Texas A&M University, 3137 TAMU, College Station, TX 77843-3137, USA; chanam@tamu.edu; 3 Department of Childhood Studies, Rutgers University, 405-7 Cooper Street, Camden, NJ 08102, USA; w.lu@rutgers.edu; 4 Department of Pediatrics, University of Washington, 1959 NE Pacific St, Seattle, WA 98195, USA; jason.mendoza@seattlechildrens.org; 5 Seattle Children’s Research Institute, 1900 9th Ave, Seattle, WA 98101, USA

**Keywords:** walking to school, child and parent self-efficacy, attitude, behavior, environment

## Abstract

As a critical social cognitive construct, self-efficacy plays a determinant role in children’s walking to school (WTS). However, little is known about factors that are underlying children’s and parents’ self-efficacy in WTS. The purpose of this study is to examine behavioral, attitudinal, and environmental correlates of child self-efficacy and parent self-efficacy in WTS, and to assess differences in the correlates of child versus parent self-efficacy. Data were collected from students (*N* = 1224) and parents (*N* = 1205) from 81 elementary schools across Texas in 2009–2012. Binary logistic regressions were conducted to identify significant factors that are associated with children’s self-efficacy and parents’ self-efficacy. Results from this study showed that the parent self-efficacy was more likely to be related to their own behaviors or attitudes, rather than the environmental factors or their child’s input. The child self-efficacy, however, was influenced not only by their own and parental behaviors or attitudes, but also by environmental factors. This study suggests that both parental and child self-efficacy are important factors to be considered when making decisions about school transportation.

## 1. Introduction

Walking to school (WTS) can help to increase children’s physical activity levels [1], and may reduce their excess weight by increasing energy expenditure [2,3]. For example, Mendoza et al. conducted a cluster randomized controlled trial of the Walking School Bus program in Texas and reported significant increases of daily moderate-to-vigorous physical activity among the intervention students when compared with the control students [3]. Research has also shown a negative relationship between WTS and children’s body mass index [4]. Despite the health benefits of WTS, the percentage of children who walk or bike to school has declined dramatically in the United States (US) over the past few decades, from 47.7% in 1969 to 12.7% in 2009 [5]. In 2010, the White House Task Force on Childhood Obesity recognized increasing the percentage of children aged 5–18 taking active transportation to school by 50% as an important benchmark towards addressing the childhood obesity problem in the US [6].

Research on WTS has expanded in the past decade, and a growing body of literature has identified numerous personal and environmental correlates of WTS, including parents’ and children’s social demographics, peer influence, parental work patterns, distance, weather conditions, traffic safety, neighborhood design, and crime [7,8,9,10]. Empirical evidence from these studies has helped the establishment of the federally supported Safe Routes to School program in the US. This program is multi-faceted supporting both programs/planning and infrastructure improvement projects [11]. Unfortunately, previous emphasis placed on environmental improvements alone has proved insufficient in most cases [12], which necessitates investigations of the other domains in the socio-ecological framework, in particular individual factors such as self-efficacy.

As a social cognitive construct, self-efficacy refers to people’s self-belief in their abilities to control their functioning, overcome difficulties, and perform specific tasks. Self-efficacy determines how people feel, think, motivate themselves, and behave [13]. In health promotion, self-efficacy has been shown to be a powerful predictor and mediator of people’s health behaviors, including physical exercise, smoking cessation, contraceptive behavior, and weight loss [14]. Several empirical studies have identified that children and adolescents with higher self-efficacy are more likely to be engaged in daily physical activity [15,16,17]. A recent study further confirmed the determining role of self-efficacy in adopting and maintaining children’s active commuting behavior, showing that both parental and child self-efficacy were positively associated with children’s WTS [18].

Despite the critical role that self-efficacy plays in promoting children’s WTS, little is known about factors that are contributing to parent and child self-efficacy. Previous studies in health behavior research have examined the sources of self-efficacy, including previous experiences or behaviors, psychological states, persuasion, and barriers [19,20,21]. However, to our knowledge, no study has explored the predictors of self-efficacy in WTS. Without the knowledge of self-efficacy correlates, effective cognitive intervention strategies for promoting self-efficacy will not be possible. Therefore, the present study aims to examine behavioral, attitudinal, and environmental correlates of child and parent self-efficacy in WTS. Further, this study assesses differences in these correlates between low and high self-efficacy groups.

## 2. Methods

This study used part of the data from the Texas Childhood Obesity Prevention Policy Evaluation (T-COPPE) project, which evaluated the implementation of two key childhood obesity prevention policies in Texas: the Safe Routes to School (SRTS) program and the Women, Infants and Children (WIC) nutrition program. This study used the survey data from the SRTS program of the T-COPPE project. The surveys conducted for the SRTS evaluation were collected from the 4th grade students and their parents from 81 elementary schools across Texas. The current cross-sectional study used the baseline child-parent survey data collected in 2009 and the follow-up child-parent survey data collected in 2012.

All of the study protocols were approved by the institutional review board at The University of Texas Health Science Center at Houston (UTHealth, HSC-SPH-08-0335). The surveys included information of personal, attitudinal, behavioral, and environmental perceptions. The built environmental measures that were used in this study were generated using Geographic Information System (GIS, Esri, Redlands, CA, USA) in 2011–2012. More information about the study instruments and methods can be found elsewhere [22,23,24].

### 2.1. Sample Selection

**Figure 1:**
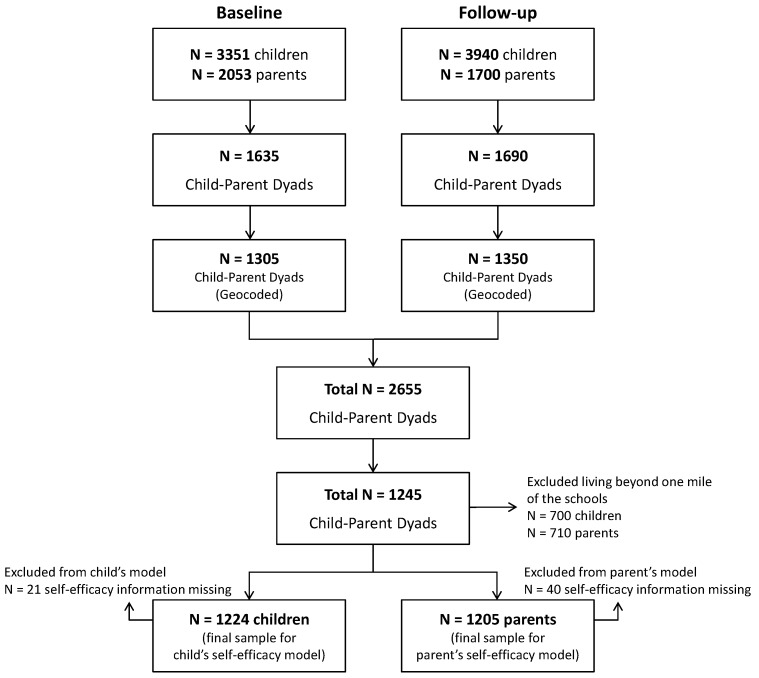
Sample Selection Process.

Figure 1 shows the process of sample selection in this study. The 4th grade students and their parents (*N* = 6500 pairs) from the approved schools were invited to complete the baseline and follow-up surveys. Of the 6500 dyads, 2053 (31.6%) parents and 3351 (51.6%) students returned their surveys at baseline, and 1700 (26.2%) parents and 3940 (60.6%) students returned their surveys at follow-up. There were 1635 child-parent survey dyads at baseline and 1690 dyads at follow-up. Of those, 1305 of the baseline and 1350 of the follow-up respondents’ residential addresses were successfully geocoded. The remaining cases were not geocoded due to missing or incomplete address information. Out of the 2655 child-parent dyads combining the baseline and the follow-up participants, only those that were living within one mile of the schools (53.1%, 1245 out of 2655) were selected for this study. Because walking is generally considered not viable for children’s school commuting beyond one mile [5,25,26], those living beyond this distance were excluded from this study aimed at evaluating the roles of self-efficacy in WTS. Further, 21 children and 40 parents who gave no information on their self-efficacy were excluded, leaving a total of 1224 children and 1205 parents to be included in our analysis.

### 2.2. Measures

#### 2.2.1. Self-Efficacy

Students and parents reported the level of self-efficacy on the following questions. For students, the survey asked “You can walk to or from school even if” and for parents, “You can allow your 4th grade child to walk to or from school even if” (a) I live far from school, (b) there is a lot of traffic, (c) it is hot outside, (d) it is cold outside, (e) it is raining outside, and (f) other children do not walk to school. The answers to these questions were: not sure, a little sure, and very sure. Based on the frequency of the participants’ responses to these six items, two dichotomous self-efficacy variables were generated for the child and parent groups. Those who answered “not sure” to all six items were coded as zero, representing “low self-efficacy”, and the rest were coded as one representing “high self-efficacy”. This “low” vs. “high” self-efficacy variable for each student and parent group was used as the main outcome of this study. The child’s school travel modes were reported by the parents as the mode used on most days among walking, biking, riding the school bus, family vehicle, and other (carpool, transit, etc.).

#### 2.2.2. Socio-Demographics

Data on student gender, race/ethnicity, parental education level, car ownership, and the number of children living in the household and attending elementary schools were collected in the parent survey. Given the sample variations of study variables, race/ethnicity was categorized as non-Hispanic White, Hispanic, and others, and parental education was dichotomized as a binary variable: low (high school or below) and high (associate degree or above).

#### 2.2.3. Personal Attitudes and Behaviors

Although both parental and child surveys included questions about attitudes and behaviors regarding WTS, the parental survey included more and detailed items. The survey questions on attitudes and behaviors included travel time to school, cues to action on WTS, agreement for participating in WTS, and outcome expectation or walking benefits acquired from WTS. The response options were recoded as necessary for analyses based on the response distribution.

#### 2.2.4. Environmental Perceptions

Both parents’ and children’s perceptions of the neighborhood environment were assessed. First, children specified their perceptions of people walking or biking in their neighborhood, adults doing physical activities or exercising in their neighborhood, and their friends who usually walk or ride a bike to school. These questions were combined to create a variable that indicated the extent to which children observed physical activities of people around them. The scores on this scale variable ranged from 0 to 4, with 0 for no physical activity and 4 for a high level of physical activity observed around children’s neighborhoods in terms of frequency. Second, children’s perceptions about whether or not there were playgrounds or parks close to their home were included. Third, parents were asked about the presence of sidewalks on the streets near their child’s school. Fourth, parents reported on the sidewalk maintenance condition, whether the sidewalks were well-paved, even, and did not have a lot of cracks. The third and the fourth survey questions included the following three response categories: “no”, “yes, a few”, or “yes, many”.

#### 2.2.5. Built Environments

Objectively measured built environmental variables were generated in 2010–2012 using ArcGIS and ESRI Business Analyst. The raw GIS data layers were provided by the State Department of Public Safety (crash incidents), the Texas Department of Transportation (road attribute information), and the Texas Natural Resources Information System (digital elevation model). The respondents’ home and school locations were geocoded first in GIS. Through the network analysis in ArcCatalog, home-to-school (HTS) routes were created, assuming that most of the children would use the shortest route to school. A comparison between the shortest and the actual routes, using the Global Positioning System data collected from a similar study population (*N* = 112) in Austin, Texas, showed about 89% matching rate. All of the built environmental variables were measured within 200 feet of the HTS route (HTS route buffer) used as appropriate buffer size to capture the environmental proximal to HTS route [27].

Built environmental variables used for this study included land use, slope, and safety. First, the total number of non-residential land uses extracted from the Business Analyst in ArcGIS was used to depict the general land use and traffic conditions. Second, slope/terrain of the HTS route buffer area was measured as the percentage of the buffer area that is steep (defined as a slope greater than 5%). Third, the number of crashes occurred within the HTS buffer from 2006 to 2009 was used to capture traffic-related safety conditions. Other safety related data were not available at the spatially disaggregated level required for this study.

### 2.3. Data Analysis

To compare the frequency or the mean of the study variables between low and high self-efficacy groups, chi-square tests were conducted for categorical variables and *t*-tests for continuous variables. With the outcome variable of self-efficacy captured as a binary scheme (0: low self-efficacy, 1: high self-efficacy), two binary logistic regression models were estimated to further identify the significant predictors of children’s self-efficacy and parents’ self-efficacy. The potential school-level clustering effect was tested using mixed effects logistic regression models, and was confirmed to be insignificant (*p* = 0.486 for children’s self-efficacy, *p* = 1.000 for parents’ self-efficacy). All of the analyses were undertaken using STATA version 14. (StataCorp LP, College Station, TX, USA).

## 3. Results

### 3.1. Descriptive Statistics

**Figure 2:**
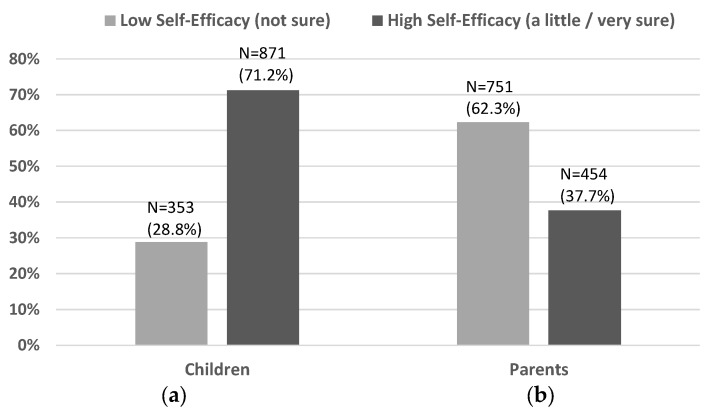
Low vs. High Self-Efficacy among Children and Parents: (**a**) Child Self-Efficacy; and, (**b**) Parent Self-Efficacy.

About 62.3% (*N* = 751 out of 1205) of the parents had low self-efficacy and 37.7% with high self-efficacy (Figure 2). In contrast, the majority (71.2%, *N* = 871 out of 1224) of the children showed high self-efficacy, while only about 28.8% (*N* = 353) had low self-efficacy. The child gender was balanced with 608 (48.8%) boys and 637 (51.2%) girls. Over half of the child samples were Hispanic (769, 62.2%), followed by non-Hispanic White (261, 21.1%) and others (207, 16.7%). A small number (<5%) of the respondent families had no vehicle, while about 40% had one vehicle, and over half had two or more vehicles. Many households had two (516, 42.2%) or more (224, 18.3%) children attending elementary school, and over two thirds (744, 68.5%) of the parents had a high school or lower level of education.

### 3.2. Bivariate Tests

#### 3.2.1. Socio-Demographic Variables

Table 1 shows comparison results of the sample characteristics between low self-efficacy and high self-efficacy groups for both children and parents. In our sample, student gender and household car ownership showed significant differences between the high and low efficacy group. Although boys and girls were evenly represented in this study, the number of girls in the low self-efficacy groups was higher than in the high self-efficacy groups. The number of carless households was higher among high parent self-efficacy group.

#### 3.2.2. Self-Efficacy and Walking to or from School

Walking to or from school was significantly different by the level of self-efficacy for both children and parents (Table 2). The percentage of walkers was higher for the high self-efficacy group than for the low self-efficacy group. Regardless of self-efficacy, the percentage of walkers was higher for children leaving school than arriving at school, and it was associated with lower family car use and higher school bus use. Furthermore, Table 2 shows that travel modes of arriving at school and of leaving school vary depending upon the self-efficacy level of both child and parent groups.

#### 3.2.3. Attitudes/Behaviors, Environmental Perceptions, and Built Environments

Table 3 presents statistical differences in the frequencies or the means of the study variables between the low and the high self-efficacy groups of children and parents. Parents with high self-efficacy had a more encouraging attitude toward their children’s walking or biking to school, and likewise, children with high self-efficacy had parents with more encouraging attitudes. Parents in the high self-efficacy group were more likely to allow their child to walk or bike to school than parents in the low self-efficacy group; similarly, children in the high self-efficacy group had parents who were more likely to allow them to walk or bike. More parent and child respondents in the high self-efficacy groups had short (<5 min) travel time to school, while higher number of respondents in the low self-efficacy groups had long (≥10 min) travel time. Children who sometimes or always asked their parents to walk or bike to school were more likely to have high self-efficacy than children who never asked. Further, outcome expectations from walking or biking to school were also higher for both parents and children in the high self-efficacy groups when compared with the low self-efficacy groups.

Children and parents who had high self-efficacy perceived seeing more adults doing physical activities in their neighborhoods. Children and parents who reported having playgrounds or parks close to their homes were more likely to have high self-efficacy, compared to those who reported not having those. Children’s self-efficacy and parents’ self-efficacy were also higher if parents perceived many well-maintained sidewalks in their neighborhoods. Among the objectively measured environmental variables, the number of crashes that were measured along the HTS routes buffer was higher for parents who had high self-efficacy than those who had low self-efficacy.

### 3.3. Multivariable Analyses

Table 4 presents the results from the binomial logistic regressions estimating the attitudinal, behavioral, and environmental correlates of self-efficacy among children and parents.

#### 3.3.1. Attitudinal and Behavioral Variables

Compared to children who never asked, children who sometimes (OR = 2.22, *p* < 0.001) or always asked (OR = 1.66, *p* = 0.055) their parents to walk to school were more likely to have high self-efficacy. Parental encouragement on children’s walking or biking to school was a positive correlate of having high self-efficacy among parents. Parents’ permission for their children’s WTS also showed a positive relationship with their own self-efficacy (OR = 1.63, *p* = 0.006). Walking benefits variable was positively associated with the parent’s self-efficacy (OR = 2.05, *p* < 0.001), while longer travel time school was associated with decreased child self-efficacy only (OR = 0.62 for more than 10 min, *p* = 0.028). The odds of having higher self-efficacy among children (OR = 2.23, *p* < 0.001) and parents (OR = 2.85, *p* < 0.001) increased if parents would allow their child to walk or bike to school. If children walked to school, their parents were more likely to have high self-efficacy (OR = 1.94, *p* = 0.007).

#### 3.3.2. Environmental Perceptions

Children were more likely to have high self-efficacy if they observed many people walking, biking, or exercising in their neighborhoods (OR = 1.35, *p* = 0.009). Furthermore, parental observation of people engaging in physical activities in neighborhood is positively associated with increased odds of having high self-efficacy among children (OR = 1.73, *p* = 0.039). Children’s perception of the presence of playgrounds or parks close to their homes further increased their odds of having high self-efficacy (OR = 1.40, *p* = 0.054). As compared to parents who never saw well-maintained sidewalks around their neighborhoods, parents who perceived having many well-maintained sidewalks showed higher level of self-efficacy (OR = 1.45, *p* = 0.063).

#### 3.3.3. Objectively-Measured Built Environments

Increase in the number of destination land uses (e.g., retail, services, entertainment, and employment) along the HTS route was positively associated with parent self-efficacy (OR = 1.02, *p* = 0.034). When compared to the absence of steep slope, the presence of steep slope in <50% of the HTS route buffer area was associated with lower parental self-efficacy (OR = 0.64, *p* = 0.028). A higher number of crashes that were measured within the HTS route buffer showed an inverse relationship with children’s self-efficacy (OR = 0.92, *p* = 0.009).

## 4. Discussion

The findings from the bivariate tests revealed a general pattern of more favorable attitudes toward WTS, better understanding of walking benefits, and more frequent walking behaviors among parents and children with high self-efficacy. The high self-efficacy groups also perceived more supportive built and social environments. For example, children and parents with high self-efficacy perceived more people walking, biking, and exercising in neighborhoods, and more playgrounds or parks close to their homes than those with low self-efficacy. Well-maintained sidewalks in the neighborhood were better perceived by children and parents with high self-efficacy. Furthermore, higher number of crash incidents was observed along the HTS route buffer for parents who had high self-efficacy. The reason may be associated with the likelihood that parents who had high self-efficacy for their children’s WTS are more likely to be aware of the benefits of walking, and to engage in walking/bicycling in walkable neighborhoods that typically have high street connectivity (representing street patterns with many intersections where the majority of crashes occur) [28]. Indeed, the bivariate test results from this study represented that children and parents with high self-efficacy were more likely to perceive the presence of many people walking, biking, and exercising in their neighborhoods, which may indicate that they live in walkable neighborhoods [29].

The results from the multivariable logistic regressions demonstrated that child and parent self-efficacy were differently associated with the study variables. Given the positive relationship between parents’ self-efficacy and children’s WTS, as also revealed by Mendoza (2010) [30], our findings suggest the need for implementing educational programs to change parents’ attitudes and behaviors to promote children’s WTS. Further, locating more destination land uses along WTS routes might help to increase parents’ self-efficacy. This finding is similar to the result from a previous study showing that land use mix increases children’s WTS [31]. However, other studies reported inconsistent findings regarding the role of land uses in promoting WTS [32,33]. Therefore, to further delineate the potential mediating effect of parent self-efficacy between land uses and children’s WTS, future studies using structural equation modeling are warranted.

In addition to the parents’ self-efficacy, specifying the ways to promote children’s self-efficacy is also important because children who have lower levels of self-efficacy for overcoming barriers or competing activities are more likely to be physically inactive in their home [34]. Our findings demonstrate that seeing other people being active in children’s neighborhoods, and having playgrounds or parks close to their home, were positively associated with children’s self-efficacy. These factors may increase children’s abilities to overcome barriers for WTS, because they may increase perceptions of safety or reflect a social norm. Further, our study reveals an inverse relationship between crash incidents along the HTS routes and children’s self-efficacy, which underscores the importance of providing children with safe environments in order to improve their self-efficacy and encourage their WTS behavior. Third, children with a high self-efficacy level perceived more people walking, biking, and exercising in neighborhoods, suggesting that child self-efficacy may be promoted by increased exposure to supportive role models in the neighborhood and positive peer influences. Interventions such as the Walking School Bus program, in which a group of students walking to/from school with adults, can be effective in promoting children’s WTS by increasing their self-motivation and self-efficacy [35].

This study has several limitations. First, as a cross sectional study, it cannot establish any causal relationship between the study variables and children’s and parents’ self-efficacy. Second, we tested multiple associations in the study, but did not do multilevel testing because we consider this study exploratory. Third, the potential for omitted variables and for interactions among the study variables exist yet not fully assessed. Nevertheless, this study is one of the first to examine predictors of self-efficacy toward children’s WTS, offering several new insights and perspectives on WTS promotion. One of the key strengths of this study is the use of both objective and self-reported measures. Additionally, we recruited participants that were representing diverse socioeconomic and demographic characteristics and from different areas of Texas, and therefore our findings have reasonable generalizability.

## 5. Conclusions

In this study, self-efficacy, as reported by children and parents, was used to compare their attitudinal, behavioral, and environmental correlates. The findings suggested self-efficacy as an important factor that requires further considerations in the school transportation decision-making process. Our study further showed that children’s and parents’ attitudes towards WTS were interdependent. In agreement with previous studies showing positive relationships between children’s self-efficacy and parents’ self-efficacy [18], this study identified that parents with high self-efficacy had more encouraging and allowing attitudes towards their children’s WTS, and likewise, children with high self-efficacy were more likely to seek parental permission for WTS. This study also suggested that both the children’s and parents’ self-efficacy might play significant roles in children’s WTS, as shown by the significantly higher percentage of WTS among the high self-efficacy groups when compared to the low self-efficacy groups. Furthermore, consistent with the previous WTS studies showing the stronger role of parents’ self-efficacy as compared to children’s self-efficacy [2,30], this study identified that children’s WTS was significantly associated with parental self-efficacy only. Nevertheless, children’s self-efficacy might hold some potential to indirectly influence WTS behavior, as this study found a positive relationship between children’s self-efficacy and parents’ self-efficacy on WTS. A previous study did report a significant correlation between children’s self-efficacy and their active commuting to school behavior [18]. Our study adds to the recommendation that both children’s self-efficacy and parents’ self-efficacy be considered in future WTS studies and intervention efforts for promoting WTS [18].

## Figures and Tables

**Table 1:** Sample characteristics by child and parent self-efficacy and bivariate tests.

Sample Characteristics	Child Self-Efficacy (*N* = 1224)			Parent Self-Efficacy (*N* = 1205)		
	Low Self-Efficacy (*N* = 353)	High Self-Efficacy (*N* = 871)	Bivariate Tests	Low Self-Efficacy (*N* = 751)	High Self-Efficacy (*N* = 454)	Bivariate Tests
	Freq. (%)	Freq. (%)	X^2^	Freq. (%)	Freq. (%)	X^2^
Child gender						
Boys	143 (40.5%)	455 (52.2%)	13.83 **	339 (45.1%)	249 (54.9%)	10.67 **
Girls	210 (59.5%)	416 (47.8%)		412 (54.9%)	205 (45.1%)	
Child race/ethnicity						
Non-Hispanic White	66 (18.9%)	190 (21.9%)	2.25	149 (20.0%)	104 (23.1%)	2.68
Hispanic	230 (65.7%)	530 (61.2%)		478 (64.0%)	267 (59.3%)	
Other	54 (15.4%)	146 (16.9%)		120 (16.0%)	79 (17.6%)	
Car ownership						
No	12 (3.5%)	40 (4.8%)	2.56	21 (2.9%)	27 (6.3%)	7.74 *
Yes, 1	150 (44.4%)	333 (39.8%)		301 (41.5%)	176 (40.7%)	
Yes, 2+	176 (52.1%)	464 (55.4%)		404 (55.6%)	229 (53.0%)	
Number of children in your household, attending elementary school						
1	125 (36.2%)	332 (38.8%)	0.61	276 (37.7%)	179 (40.0%)	0.67
2	155 (44.9%)	358 (41.9%)		316 (43.2%)	184 (41.2%)	
3+	65 (18.8%)	164 (19.2%)		140 (19.1%)	84 (18.8%)	
Parents’ highest level of education						
High school or below	228 (72.2%)	501 (66.6%)	3.14	461 (68.9%)	256 (66.8%)	0.48
Associate degree or above	88 (27.8%)	251 (33.4%)		208 (31.1%)	127 (33.2%)	

* 0.01 ≤ *p* < 0.05; ** *p* < 0.01.

**Table 2:** Travel modes by child and parent self-efficacy and bivariate tests.

Sample Characteristics	Child Self-Efficacy (*N* = 1224)			Parent Self-Efficacy (*N* = 1205)		
	Low Self-Efficacy (*N* = 353)	High Self-Efficacy (*N* = 871)	Bivariate Tests	Low Self-Efficacy (*N* = 751)	High Self-Efficacy (*N* = 454)	Bivariate Tests
	Freq. (%)	Freq. (%)	X^2^	Freq. (%)	Freq. (%)	X^2^
Walking to or from school						
Non-walk	303 (92.9%)	624 (75.8%)	43.93 **	656 (92.7%)	252 (59.7%)	181.73 **
Walk	23 (7.1%)	199 (24.2%)		52 (7.3%)	170 (40.3%)	
Travel modes for arriving at school						
Walk	13 (3.8%)	112 (13.4%)	35.96 **	25 (3.4%)	98 (23.0%)	145.20 **
Bike	1 (0.3%)	19 (2.3%)		3 (0.4%)	16 (3.8%)	
School bus	64 (18.9%)	101 (12.0%)		132 (18.1%)	29 (6.8%)	
Family car	252 (74.3%)	578 (68.9%)		545 (74.6%)	273 (63.9%)	
Other	9 (2.7%)	29 (3.4%)		26 (3.5%)	11 (2.5%)	
Travel modes for leaving school						
Walk	17 (5.2%)	179 (22.5%)	70.35 **	42 (6.1%)	156 (38.2%)	214.79 **
Bike	1 (0.3%)	19 (2.4%)		3 (0.4%)	16 (3.9%)	
School bus	98 (30.3%)	130 (16.4%)		178 (25.8%)	46 (11.3%)	
Family car	193 (59.6%)	418 (52.6%)		423 (61.2%)	175 (42.9%)	
Other	15 (4.6%)	48 (6.1%)		45 (6.5%)	15 (3.7%)	

** *p* < 0.01 level.

**Table 3:** Descriptive statistics and bivariate tests for attitudinal, behavioral, and environmental variables.

Sample Characteristics	Child Self-Efficacy (*N* = 1224)			Parent Self-Efficacy (*N* = 1205)		
	Low Self-Efficacy (*N* = 353)	High Self-Efficacy (*N* = 871)	Bivariate Tests	Low Self-Efficacy (*N* = 751)	High Self-Efficacy (*N* = 454)	Bivariate Tests
	Freq. (%) or Mean ± SD	Freq. (%) or Mean ± SD	X^2^ or T	Freq. (%) or Mean ± SD	Freq. (%) or Mean ± SD	X^2^ or T
**Attitudes and Behaviors**						
I encourage my child to walk or bike to school. (P)						
Never	275 (78.1%)	459 (53.0%)	68.44 ^a,^**	580 (77.4%)	144 (31.8%)	282.36 ^a,^**
Not very often	36 (10.2%)	146 (16.8%)		94 (12.6%)	84 (18.5%)	
Yes, some/all of time	41 (11.7%)	262 (30.2%)		75 (10.0%)	225 (49.7%)	
I would allow my child to walk or bike. (P)						
No	209 (60.6%)	262 (31.0%)	89.87 ^a,^**	395 (53.5%)	64 (14.5%)	177.30 ^a,^**
Yes	136 (39.4%)	584 (69.0%)		343 (46.5%)	378 (85.5%)	
On most days how long does it take your child to get to school? (P)						
<5 min	96 (28.8%)	331 (40.2%)	18.47 ^a,^**	237 (33.5%)	195 (45.0%)	22.66 ^a,^**
5–10 min	124 (37.3%)	301 (36.5%)		254 (35.9%)	155 (35.8%)	
≥10 min	113 (33.9%)	192 (23.3%)		216 (30.6%)	83 (19.2%)	
Walking benefits (outcome expectation) (P)						
Scale (1–3)	1.92 ± 0.64	2.09 ± 0.62	17.32 ^b,^**	1.87 ± 0.62	2.31 ± 0.53	157.65 ^b,^**
Has your child asked for permission to walk to or from school in the last year? (P)						
No	230 (67.6%)	358 (42.4%)	61.98 ^a,^**	446 (61.4%)	128 (28.8%)	116.65 ^a,^**
Yes	110 (32.4%)	487 (57.6%)		281 (38.6%)	316 (71.2%)	
How often do you ask your parents if you can walk to school? (S)						
Never ask	255 (75.0%)	372 (54.3%)	40.96 ^a,^**	453 (65.2%)	163 (52.6%)	14.59 ^a,^**
Sometimes ask	53 (15.6%)	195 (28.5%)		149 (21.4%)	94 (30.3%)	
Always ask	32 (9.4%)	118 (17.2%)		93 (13.4%)	53 (17.1%)	
**Parent and Student Environmental Perceptions**						
People walk, bike, or exercise in neighborhood (P)						
No	49 (14.4%)	83 (9.7%)	10.64 ^a,^**	91 (12.4%)	38 (8.5%)	15.28 ^a,^**
Yes, a few	201 (58.9%)	473 (55.3%)		430 (58.6%)	231 (51.9%)	
Yes, many	91 (26.7%)	300 (35.0%)		213 (29.0%)	176 (39.6%)	
Sidewalks in your neighborhood are well maintained (paved, even, and not a lot of cracks) (P)						
No	141 (43.9%)	263 (33.7%)	11.16 ^a,^*	290 (42.8%)	112 (26.9%)	32.07 ^a,^**
Yes, a few	96 (29.9%)	253 (32.4%)		206 (30.4%)	139 (33.4%)	
Yes, many	84 (26.2%)	264 (33.9%)		181 (26.7%)	165 (39.7%)	
People walk, bike, or exercise in neighborhood (S)						
Scale (0–3.67)	1.42 ± 0.72	1.71 ± 0.76	39.26 ^b,^**	1.56 ± 0.75	1.72 ± 0.77	12.69 ^b,^**
There are playgrounds or parks close to your home (S)						
No	114 (38.5%)	197 (25.1%)	18.88 ^a,^**	208 (31.7%)	101 (24.7%)	5.83 ^a,^**
Yes	182 (61.5%)	588 (74.9%)		449 (68.3%)	307 (75.3%)	
**Objective Environmental Variables**						
Number of destination land uses						
Scale (1–31)	5.41 ± 11.06	4.95 ± 9.27	0.70 ^b^	5.03 ± 8.68	5.06 ± 10.75	0.05 ^b^
Steep slopes > 5% (Ref. 0%)						
0%	133 (37.7%)	289 (33.2%)	3.17 ^a^	251 (33.4%)	163 (35.9%)	3.88 ^a^
0.1 ≤ 50%	132 (37.4%)	327 (37.5%)		302 (40.2%)	157 (34.6%)	
50.1 ≤ 100%	88 (24.9%)	255 (29.3%)		198 (26.4%)	134 (29.5%)	
Number of crashes (continuous variable)						
Range: 0–19	1.57 ± 2.78	1.48 ± 2.28	0.30 ^b^	1.42 ± 2.48	1.58 ± 2.28	1.26 ^b,^*

^a^ chi-square test, ^b^ *t*-test; ** *p* < 0.01 level, * 0.01 ≤ *p* < 0.05; (P): parent survey questions, (S): student survey questions.

**Table 4:** Fully adjusted models estimating the odds of having “high self-efficacy”.

Variable Description	Child Self-Efficacy (*N* = 802, Pseudo R^2^ = 0.1029)			Parent Self-Efficacy (*N* = 994, Pseudo R^2^ = 0.2736)		
	O.R.	95% C.I.		O.R.	95% C.I.	
		Lower	Upper		Lower	Upper
**Socio-Demographics**						
Child Gender (Ref. Boy)						
Girl	0.64 ***	0.47	0.89	0.83	0.60	1.16
Car ownership (Ref. No car ownership)						
Yes, 1	-	-	-	0.42 **	0.18	0.96
Yes, 2+	-	-	-	0.49 *	0.22	1.10
**Attitude and Behaviors**						
I encourage my child to walk or bike to school. (P) (Ref. Never)						
Not very often	-	-	-	1.85 ***	1.19	2.87
Yes, some/all of the time	-	-	-	3.85 ***	2.39	6.19
I would allow my child to walk or bike. (P) (Ref. No)						
Yes	2.23 ***	1.61	3.08	2.85 ***	1.93	4.21
On most days how long does it take child to get to school? (P) (Ref. Less than 5 min)						
5–10 min	0.74	0.50	1.09	-	-	-
More than 10 min	0.60 **	0.40	0.92	-	-	-
How often do you ask your parents if you can walk to school? (S) (Ref. Never ask)						
Sometimes ask	2.22 ***	1.47	3.35	-	-	-
Always ask	1.66 *	0.99	2.81	-	-	-
Walking benefits (P) (Outcome expectation)						
Scale (1–3)	-	-	-	2.05 ***	1.54	2.74
Has your child asked for permission to walk to or from school in the last year? (P) (Ref. No)						
Yes	-	-	-	1.63 ***	1.15	2.30
Walking to school (Ref. No)						
Yes	-	-	-	1.94 ***	1.20	3.12
**Environmental Perceptions**						
People walk, bike, or exercise in neighborhood (S)						
Scale (0–4)	1.35 **	1.07	1.70	-	-	-
People walk, bike, or exercise in neighborhood (P) (Ref. No)						
Yes, a few	1.40	0.85	2.32	-	-	-
Yes, many	1.73 *	1.00	2.99	-	-	-
There are playgrounds or parks close to your home (S) (Ref. No)						
Yes	1.39 *	0.99	1.96	-	-	-
Sidewalks in your neighborhood are well maintained (paved, even, and not a lot of cracks) (P) (Ref. No)						
Yes, a few	-	-	-	1.15	0.77	1.74
Yes, many	-	-	-	1.45 *	0.98	2.16
**Objective Environments**						
Total number of destination land uses within HTS route buffer						
Range: 0–118	-	-	-	1.02 **	0.49	1.05
Steep slopes > 5% within HTS route buffer (Ref. 0%)						
0.1 ≤ 50%	-	-	-	0.64 **	0.43	0.95
50.1 ≤ 100%	-	-	-	1.02	0.66	1.57
Total number of crashes within HTS route buffer						
Range: 0–118	0.93 **	0.87	0.98	-	-	-

(S): student survey questions, (P): parent survey questions; O.R.: odds ratio, C.I.: confidence interval; * *p* < 0.10, ** *p* < 0.05, *** *p* < 0.01 level.
